# Fear of childbirth and its determinants in pregnant women in the third trimester: a cross-sectional study

**DOI:** 10.1186/s12888-023-05070-7

**Published:** 2023-08-08

**Authors:** Teng Zhang, Meilin Liu, Fanli Min, Wei Wei, Yuan Liu, Jiao Tong, Qian Meng, Lizhou Sun, Xu Chen

**Affiliations:** 1 Department of Obstetrics, Lianyungang Maternal and Child Health Hospital, 669 Qindongmen Street, Haizhou District, Lianyungang, 222000 Jiangsu P.R. China; 2 Department of Obstetrics and Gynecology, First Affiliated Hospital of Nanjing Medical University, Nanjing, Jiangsu China

**Keywords:** Fear of childbirth, Pregnant women, Associated factors, China

## Abstract

**Background:**

Fear of childbirth (FOC) is a prevalent issue among pregnant women and significantly relates to adverse outcomes for the mother and child. However, it is not clear the prevalence and risk factors of FOC among pregnant women in a region with a moderate level of economic development in China. The aim of this study was to investigate the prevalence and risk factors of FOC among pregnant women in the third trimester of pregnancy in Lianyungang city, Eastern China.

**Methods:**

A cross-sectional survey was conducted from December 2022 to February 2023 among pregnant women in the third trimester who met the inclusion criteria and visited Lianyungang Maternal and Child Health Hospital in Jiangsu Province, Eastern China. A structured questionnaire including sociodemographic characteristics, clinical characteristics, FOC, family function, doctor-patient communication, social support, general self-efficacy, anxiety, depression, insomnia symptoms, and quality of life was used to collect data. A multiple linear regression model was used to identify predictors of FOC.

**Results:**

This study included 535 pregnant women in the third trimester. The mean score of FOC was 30.67 ± 10.18, and the median score was 29.00. The prevalence of FOC was 56.64%. Multiple linear regression analysis revealed that pregnant women with electronic screen exposure time more than 5 h per day (*β* = 2.02, 95%*CI*: 0.50–3.53, *P* < 0.05), no history of cesarean section (*β* = 2.66, 95%*CI*: 0.61–4.71, *P* < 0.05), likes sour food or hates greasy food (*β* = 1.75, 95%*CI*: 0.00-3.50, *P* < 0.05), anxiety (*β* = 0.50, 95%*CI*: 0.21–0.80, *P* < 0.05) and depression (*β* = 0.30, 95%*CI*: 0.04–0.57, *P* < 0.05) were more likely to have a greater level of FOC than their counterparts. However, a significantly lower level of FOC was observed in pregnant women who were multipara (*β*=-1.64, 95%*CI*: -3.27–0.01, *P* < 0.05), not worrying about delivery without family members (*β*=-3.75, 95%*CI*: -5.26–2.25, *P* < 0.001), had good family function (*β*=-0.32, 95%*CI*: -0.64–0.00, *P* < 0.05) and doctor-patient communication (*β*=-0.33, 95%*CI*: -0.64–0.02, *P* < 0.05).

**Conclusions:**

The prevalence of FOC was high in Lianyungang city, Eastern China. FOC is influenced by multiple factors. There is an urgent need to develop interventions to reduce the prevalence of FOC in the third trimester of pregnancy, and to pay attention to pregnant women with risk factors for FOC.

## Background

Childbirth is a natural process, but it is also an unpredictable and painful one that can even cause the death of the mother and baby. During pregnancy, pregnant women experience a range of physical and mental changes, including fear of childbirth [1]. Fear of childbirth (FOC) includes both excessive maternal preoccupation with labor pain during pregnancy [2] and secondary fear of childbirth following normal delivery, miscarriage, and termination of pregnancy [3–5]. The prevalence of FOC varies from country to country and region to region, ranging from 4–82% [6–9], and has shown an increasing trend recently [6], which has gradually attracted great attention from the academic community.

FOC can have adverse outcomes for the mother and child. During pregnancy, FOC may cause changes in the uterine environment, with implications for increased fetal heart rate and reduced intrauterine movement [10], which are associated with the increased risk of postdates, intrauterine growth restriction and fetal distress as signs of fetal hypoxemia [11]. FOC may also increase the risk of adverse neonatal outcomes such as preterm childbirth and low birth weight [12]. Moreover, FOC are associated with prolonged labor, cesarean section, choice of epidural analgesia, prenatal and postpartum depression, and anxiety [13, 14]. In addition, FOC can increase perinatal costs, and the cost of severe FOC pregnant women is significantly higher than that of mild [15]. Therefore, it is necessary to understand the prevalence and risk factors for FOC.

The risk factors for FOC are complex and multifaceted. Previous studies in Europe and the Middle East have reported a number of risk factors for FOC, including nullipara, advanced maternal age, low socioeconomic status, unplanned pregnancy, previous cesarean section, vacuum delivery, perineal tear, shoulder dystocia, pregnancy complications (e.g., gestational diabetes), anxiety, depression, low self-efficacy, low self-esteem, negatively appraising birth, loneliness, fear of pain, disagreement with the birth plan proposed by the obstetrician [16–21]. However, there are differences.

between these regions and China in terms of life background, ethnic religion, and social structure, so the findings from these regions may not be applicable to Chinese populations.

In recent years, Chinese scholars have also started to try to study risk factors for FOC in some cities, such as Xi’an, Chongqing, Changsha and Guangdong, and found that primiparas, advanced maternal age, unplanned pregnancy, few spousal support, few social support, previous cesarean section, low self-efficacy, depression and use of pregnancy-related smartphone applications were the main risk factors [9, 14, 22–24]. However, these cities are national central cities or high developed regions in China, which differ from the middle or less developed areas in terms of their living background, social structure, etc. Therefore, it is necessary to conduct cross-sectional studies to understand the prevalence of FOC and its influencing factors in the middle or less developed areas in China.

In addition to the aforementioned influencing factors, there are also some factors that may potentially increase the occurrence of FOC. For example, certain clinical symptoms (such as abdominal pain and vaginal bleeding) during pregnancy are a painful experience for pregnant women, which makes them fearful when facing a more painful upcoming delivery experience. Therefore, these clinical symptoms may be a potential risk factor for FOC. Environmental pollution increases the risk of adverse fetal/neonatal outcomes [25, 26]. When faced with an impending delivery, pregnant women may experience FOC due to excessive concerns about their child’s health. Maternal disagreement with the birth plan proposed by the obstetrician was a risk factor for severe FOC [18], which might be partly related to doctor-patient communication. Clinical symptoms (e.g., abdominal pain, vaginal bleeding), environmental pollution, and Doctor-patient communication have not received sufficient attention in previous studies on the prevalence of FOC and its influencing factors, which is lacking of analysis of these modules.

Pregnant women had more intense FOC in the third trimester than in the first or second trimester of pregnancy [3]. The third trimester is a period of particular concern. However, few studies have focused on the prevalence of FOC and its influencing factors in the third trimester of pregnancy in China. Therefore, this study was conducted in a cross-sectional study in an middle developed area, with the main objective of understanding the prevalence of FOC and providing an in-depth analysis of the effects of sociodemographic characteristics, clinical characteristics, family function, doctor-patient communication, social support, general self-efficacy, anxiety, depression, insomnia symptoms, and quality of life on FOC in the third trimester of pregnancy, in order to provide a theoretical basis for the development and implementation of targeted interventions.

## Methods

### Study design and setting

A cross-sectional survey was conducted from December 2022 to February 2023 in Lianyungang Maternal and Child Health Hospital in Jiangsu Province, Eastern China. Lianyungang Maternal and Child Health Hospital is the only tertiary maternal and child health hospital in Lianyungang, which is responsible for providing comprehensive health care services for women and children in the whole city. Lianyungang is located in the middle of China’s coast, in the northeast part of underdeveloped Jiangsu Province, covering an area of about 7,615 square kilometers. In 2021, the city’s permanent population was 4.602 million, the birth population was 31,400, and the per capita gross domestic product (GDP) was 81,015 CNY (the national per capita GDP was 80,976 CNY).

### Participants

Pregnant women in the third trimester who met the inclusion criteria and visited Lianyungang Maternal and Child Health Hospital from December 2022 to February 2023 were recruited in this study. The inclusion criteria for pregnant women included the following: (1) gestational age of 28 weeks or more; (2) age greater than or equal to 18 years; (3) no intellectual disability, cognitive impairment or major diseases; (4) able to understand the content of the questionnaire and agree to participate in this study. The exclusion criteria for pregnant women included the following: (1) pregnant women with a clearly diagnosed psychiatric disorder or a history of any psychiatric disorder; (2) unable to communicate normally; (3) do not agree to participate in this study. The recruited pregnant women independently completed a questionnaire distributed by the investigators. To ensure the quality of the data, a fixed team of 5 uniformly trained investigators collected the questionnaires in this study. Single population proportion formula was employed to calculate the minimum sample size required. Because there were no previous studies at the study sites, to get maximize the minimum sample size we used prevalence of FOC as 50% (*P* = 50%), 95%*CI*, margin error of 5%, and 20% nonresponse rate. The minimum sample size required was calculated to be 461. A systematic random sampling method was employed to select participants. In order to obtain more reliable conclusions, a total of 550 pregnant women were recruited in this study, of which 15 were excluded due to incomplete questionnaire filling due to time constraints. Therefore, a total of 535 pregnant women were included in the study, with a participation rate of 97.3%.

### Data collection

Data were collected using a structured questionnaire designed on the basis of literature review and consultation with experts. To ensure the validity of the questionnaire, a pre-survey was conducted at the study site. The questionnaire was modified and refined according to the pre-survey. The questionnaire was composed of 11 parts: sociodemographic characteristics, clinical characteristics, FOC, family function, doctor-patient communication, social support, general self-efficacy, anxiety, depression, insomnia symptoms and quality of life (QOL). Sociodemographic characteristics included age, education, area of residence, occupation, monthly family income, self-rated stress, perceived poor resistance, daily time of exposure to electronic screens, and environmental pollution around the home. Environmental pollution around the home was measured by asking “Is there any environmental pollution within 100 meters of your home, such as sewer, garbage dump, noise, heating company (fuel), etc.?”. Clinical characteristics mainly focused on parity, history of abortion, history of cesarean section, threatened abortion, complication of pregnancy, hospitalization during pregnancy, clinical symptoms (vomiting, lower abdominal pain, vaginal bleeding, dizziness and fatigue, loss of appetite, likes sour food or hates greasy food) and worrying about delivery without family members. Acid food includes acid fruit, acid dried fruit, yogurt, pickled Chinese cabbage and so on. Greasy food included greasy and fatty meat foods.

FOC was measured using the Chinese version of the Childbirth Attitude Questionnaire (CAQ) validated in Chinese pregnant women [27]. The questionnaire was originally designed by Areskog [28] and developed by Lowe [29] and Tanglakmankhong [30]. It included 16 items, and each answer of the subjects was rated on a 4-point Likert scale (1, “not at all” to 4, “high”). Item scores were summed to the total questionnaire score, which ranged from 16 to 64. Higher scores indicate more severe FOC. Those pregnant women with a score greater than or equal to 28 were considered to have FOC, with 28 to 39 classified as mild, 40 to 51 as moderate, and 52 to 64 as severe [14]. In the current study, the questionnaire had high internal consistency (Cronbach’s α = 0.95).

Family function was measured using Adaptation Partnership Growth and Resolved (APGAR) questionnaire [31]. The questionnaire consisted of 5 items, and each item was scored on a 3-point Likert scale (0, “hardly ever” to 3, “almost always”). The total score was the sum of each item score, ranging from 0 to 10. Higher scores indicated better family function. This questionnaire was widely used in the assessment of family function in pregnant women [32]. In the current study, the questionnaire had excellent internal consistency (Cronbach’s α = 0.92).

Doctor-patient communication was measured using a component derived from the Consumer Assessment of Healthcare Providers and Systems (CAHPS) [33]. This component consisted of 4 items, which related to the way medical staff explain, listen carefully, respect and time spent. The never, sometimes, usually, and always response formats were used for each item and were assigned 1, 2, 3, and 4 points, respectively. The sum of item scores was the total score of the scale, which ranged from 4 to 16. High scores reflected good doctor-patient communication. The scale had good reliability and validity [34]. In the current study, its Cronbach’s α was 0.89.

Social support was measured using the Oslo 3-item social support scale, which was frequently used to assess social support related issues in community settings [35]. The scale dealt with the number of close people on whom serious problems can be relied, the degree to which people care, and the level of ease of getting practical help from neighbors. Total scores ranged from 3 to 14, with higher scores indicating better social support [35]. In the current study, the scale had acceptable internal consistency (Cronbach’s α = 0.60).

General self-efficacy was measured using the General Self-Efficacy Scale (GSES). Self-efficacy refers to an individual’s perception or belief about whether he or she can adopt appropriate behaviors in the face of environmental challenges [36]. The GSES was a self-reported measure consisting of 10 items. Each item was scored using a 4-point Likert scale (1, “not at all true” to 4, “exactly true”). The scores of the 10 items were summed to obtain a total score, which ranged from 10 to 40. A higher total score represented a higher self-efficacy of the study subjects. The scale has been shown to have good internal consistency across different countries [37]. In the current study, its Cronbach’s α was 0.91.

Anxiety was measured using the Generalized Anxiety Disorder-7 (GAD-7) scale suitable for use in the perinatal period [38]. It was a 7-item scale with each item scored on a 4-point Likert scale (0, “not at all” to 3, “almost every day”). The total score ranged from 0 to 21, with higher scores indicating more severe anxiety symptoms. In the current study, the scale had high internal consistency (Cronbach’s α = 0.93).

Depression was measured using the Patient Health Questionnaire-9 (PHQ-9) used to screen for depressive symptoms and assess their severity [39]. The questionnaire consisted of 9 items, each of which was scored using a 4-point Likert scale (0, “not at all” to 3, “almost every day”). Total scores ranged from 0 to 27, with higher scores indicating more severe depressive symptoms. The questionnaire has been widely used in epidemiological surveys and has good reliability and validity [40]. In the current study, the questionnaire had good internal consistency (Cronbach’s α = 0.86).

Insomnia symptoms were measured using the Insomnia Severity Index (ISI), which has been shown to have good reliability and validity [41]. It consisted of 7 items, each of which was scored using a 5-point Likert scale, ranging from 0 to 4. The total score ranges from 0 to 28, with higher scores indicating more severe insomnia symptoms. In the current study, it had high internal consistency (Cronbach’s α = 0.90).

QOL was assessed using the EUROHIS-QOL 8-item index (WHOQOL-8) derived from the WHOQOL-100 and the WHOQOL-BREF [42, 43]. The scale consisted of 8 items including psychological, physical, social and environmental domains. Questions were answered based on a 5-point Likert scale, ranging from 1 to 5. The total score was the sum of 8 item scores, with higher scores indicating better QOL. The scale has been shown to have satisfactory internal consistency across multiple countries [42, 43]. In the current study, its Cronbach’s α was 0.83.

### Data analysis

All data analyses were performed using SPSS21.0 (IBM Corporation, Armonk, State of New York) software. Continuous data were described as means and standard deviations (SDs) or medians and interquartile ranges, and categorical data were described as frequencies and percentages. The rank sum test was used to compare FOC of pregnant women in the third trimester among different groups. Mann-Whitney U test was used for comparison between the two groups, and Kruskal-Wallis H test was used for comparison between the three groups. Spearman’s correlation analysis was used to assess correlations between family function, doctor-patient communication, social support, general self-efficacy, anxiety, depression, insomnia symptoms, QOL and FOC. Significant variables from univariate analyses were included in multiple linear regression models to exclude the influence of confounding factors and to identify independent related factors of FOC. All tests were two-sided, and the level of statistical significance was set to *P* < 0.05.

## Results

### Current status of FOC in pregnant women in the third trimester

**Fig. 1:**
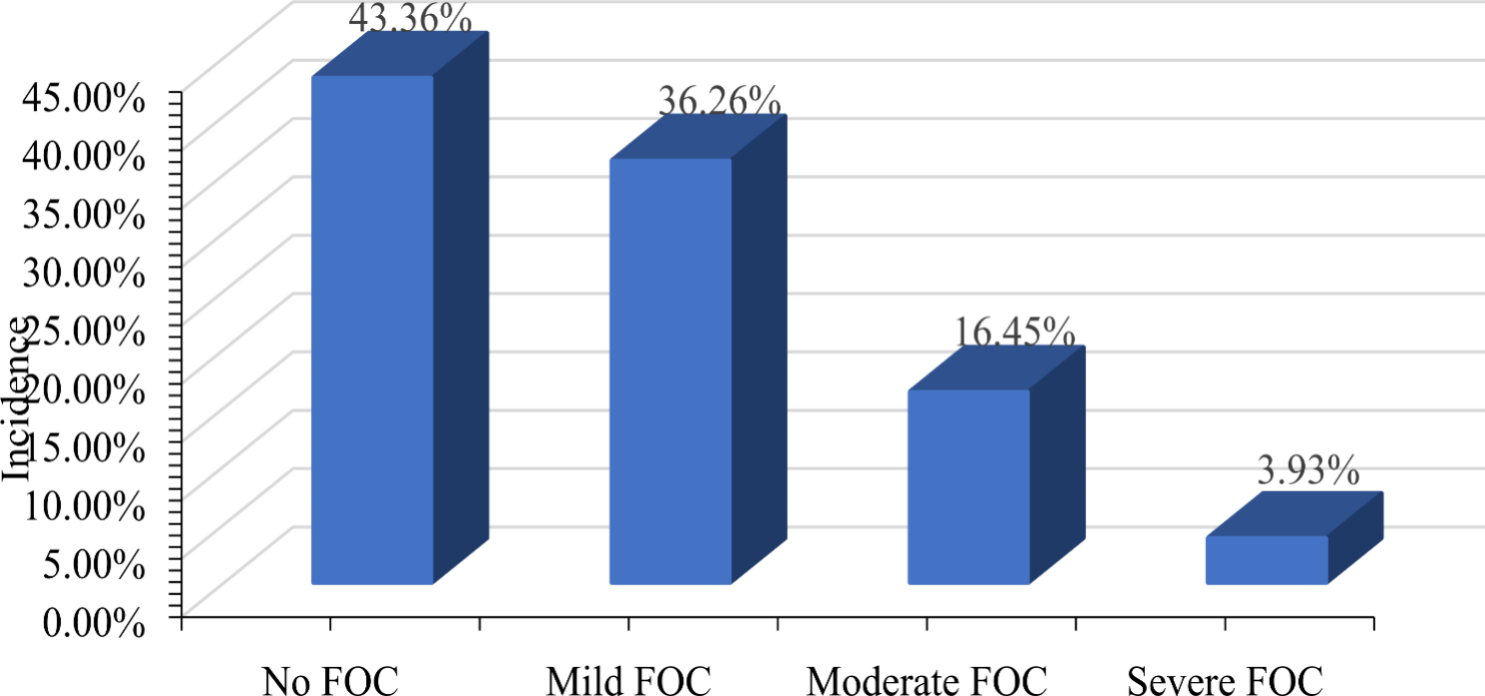
Current status of FOC in pregnant women in the third trimester. Notes: FOC: fear of childbirth

Among 535 pregnant women in the third trimester, the mean score of FOC was 30.67 ± 10.18, and the median score was 29.00. The prevalence of FOC was 56.64%, with mild 36.26%, moderate 16.45% and severe 3.93% (Fig. 1).

### Sociodemographic characteristics

The pregnant women who participated in the study ranged in age from 18 to 41 years, with a mean age of 29.39 ± 4.47 years, and a small number (12.52%) were 35 years or older. Nearly two-thirds of pregnant women (66.54%) had a college education or above, and nearly one-third (33.27%) lived in rural areas. More than half of pregnant women (53.83%) were currently employed, and the largest number (43.93%) had a family income of 5001 to 10,000 a month. Nearly a quarter (23.93%) of pregnant women self-rated their stress as big, and a smaller proportion (4.86%) perceived that they had a poor resistance. More than half of pregnant women (52.71%) were exposed to electronic screens for more than 5 h per day, and about one in five (19.81%) reported environmental pollution around their homes. The univariate analyses showed that the FOC scores of pregnant women were statistically significant among different age, education, self-rated stress, perceived poor tolerance, time of electronic screen exposure per day and environmental pollution around the home (*P* < 0.05) (Table 1).

**Table 1:** Sociodemographic characteristics and their relationships with fear of childbirth

Variables	Pregnant women			FOC		*P*
	n	%		median	interquartile range	
Age (years)						**0.040**
<35	468	87.48	29.50		24.00–37.00	
≥35	67	12.52	26.00		21.00–34.00	
Education						**0.049**
High school or below	179	33.46	28.00		21.00–36.00	
College or above	356	66.54	30.00		24.00-36.75	
Area of residence						0.293
Urban	357	66.73	30.00		24.00–36.00	
Rural	178	33.27	28.00		22.00–37.00	
Employed						0.713
Yes	288	53.83	29.00		24.00–35.00	
No	247	46.17	29.00		22.00–38.00	
Family income (RMB/month)						0.159
≤5000	100	18.69	27.50		22.25-33.00	
5001–10,000	235	43.93	30.00		23.00–37.00	
≥ 10,001	200	37.38	29.00		24.00–38.00	
Self-rated stress						**< 0.001**
Small	407	76.07	28.00		23.00–34.00	
Big	128	23.93	33.00		27.00-43.75	
Perceived poor resistance						**0.011**
Agree	26	4.86	35.50		26.50-44.25	
Disagree	509	95.14	29.00		23.00–35.00	
Time of electronic screen exposure per day (hours)						**< 0.001**
≤ 5	253	47.29	28.00		22.00-33.50	
>5	282	52.71	31.00		25.00–40.00	
Environmental pollution around the home						**< 0.001**
Yes	106	19.81	32.00		26.00–43.00	
No	429	80.19	28.00		23.00–34.00	

**Notes**: FOC: fear of childbirth; Significant values are in bold

### Clinical characteristics

Among the respondents, more than half (55.70%) were multiparous and more than one-third (34.39%) had a history of abortion. The number of pregnant women with a history of cesarean Sect. (19.44%) was approximately a quarter of those without. Similarly, the number of pregnant women with threatened abortion (18.88%) were approximately a quarter of those without. More than a quarter of pregnant women (26.36%) experienced pregnancy complications, and a large proportion (78.50%) did not experience hospital admission during pregnancy. Among the recruited pregnant women, 350 (65.42%) had vomiting during pregnancy, 78 (14.58%) had lower abdominal pain, 109 (20.37%) had vaginal bleeding, 151 (28.22%) had dizziness and fatigue, 185 (34.58%) had loss of appetite, and 147 (27.48%) had likes sour food or hates greasy food. Among the participants, nearly half of the pregnant women (45.42%) reported concerns about not having a family member with them during delivery. The univariate analyses indicated that there were statistically significant differences in FOC scores of pregnant women according to parity, history of abortion, history of cesarean section, threatened abortion, lower abdominal pain, vaginal bleeding, dizziness and fatigue, loss of appetite, likes sour food or hates greasy food and worrying about delivery without family members. (*P* < 0.05) (Table 2).

**Table 2:** Clinical characteristics and their relationships with fear of childbirth

Variables	Pregnant women			FOC		*P*	
	n	%		median	interquartile range		
Parity						**0.002**	
Nullipara	237	44.30	31.00		25.00-36.50		
Multipara	298	55.70	28.00		21.00-35.25		
History of abortion						**0.047**	
Yes	184	34.39	27.00		21.00–37.00		
No	351	65.61	30.00		25.00–36.00		
History of cesarean section						**< 0.001**	
Yes	104	19.44	25.50		19.00-33.75		
No	431	80.56	30.00		25.00–37.00		
Threatened abortion						**0.009**	
Yes	101	18.88	32.00		25.00-43.50		
No	434	81.12	29.00		23.00-34.25		
Complication of pregnancy						0.114	
Yes	141	26.36	31.00		23.50–40.50		
No	394	73.64	29.00		23.00–35.00		
Hospitalization during pregnancy						0.958	
Yes	115	21.50	29.00		25.00–35.00		
No	420	78.50	29.00		23.00–37.00		
Vomiting during pregnancy						0.791	
Yes	350	65.42	30.00		23.00-37.25		
No	185	34.58	29.00		24.00–34.00		
Lower abdominal pain						**< 0.001**	
Yes	78	14.58	32.00		26.75-44.00		
No	457	85.42	29.00		23.00–35.00		
Vaginal bleeding						**0.004**	
Yes	109	20.37	31.00		25.00–42.00		
No	426	79.63	29.00		23.00–35.00		
Dizziness and fatigue						**< 0.001**	
Yes	151	28.22	32.00		25.00–40.00		
No	384	71.78	28.00		23.00–34.00		
Loss of appetite							
Yes	185	34.58	30.00		25.00–41.00	**0.019**	
No	350	65.42	29.00		23.00–34.00		
Likes sour food or hates greasy food						**< 0.001**	
Yes	147	27.48	32.00		25.00–42.00		
No	388	72.52	28.00		22.00–34.00		
Worrying about delivery without family members						**< 0.001**	
Yes	243	45.42	31.00		26.00–41.00		
No	292	54.58	27.00		22.00–33.00		

**Notes**: FOC: fear of childbirth; Significant values are in bold

### Correlations analyses

Among the pregnant women interviewed, the median scores of family function, doctor-patient communication, social support and general self-efficacy were 9.00, 13.00, 11.00 and 28.00, respectively. The correlation analyses showed that family function, doctor-patient communication, social support and general self-efficacy were negatively correlated with FOC (*r*=-0.227, *r*=-0.132, *r*=-0.167 and *r*=-0.107, respectively, *P* < 0.05). Among participants, the median scores for anxiety, depression, and insomnia symptoms were 2.00, 5.00, and 8.00, respectively. The correlation analyses found that anxiety, depression and insomnia symptoms were positively correlated with FOC (*r* = 0.479, *r* = 0.422 and *r* = 0.294, respectively, *P* < 0.001). Among the respondents, the mean score of QOL was 29.93 ± 3.89, and the correlation analyses showed that QOL was negatively correlated with FOC (*r*=-0.251, *P* < 0.001) (Table 3).

**Table 3:** Correlation analyses and descriptive statistics of variables

Variables	Median (*P*_25_, *P*_75_)	Mean ± SD	Correlation with FOC	
			Correlation coefficients	*P*
Family function	9.00 (5.00, 10.00)		-0.227	**< 0.001**
Doctor-patient communication	13.00 (11.00, 16.00)		-0.132	**0.002**
Social support	11.00 (10.00, 12.00)		-0.167	**< 0.001**
General self-efficacy	28.00 (25.00, 30.00)		-0.107	**0.013**
Anxiety	2.00 (0.00, 6.00)		0.479	**< 0.001**
Depression	5.00 (2.00, 8.00)		0.422	**< 0.001**
Insomnia symptoms	8.00 (5.00, 12.00)		0.294	**< 0.001**
QOL		29.93 ± 3.89	-0.251	**< 0.001**

**Notes**: FOC: fear of childbirth; QOL: quality of life; Significant values are in bold

### Multiple linear regression for predicting FOC

The multiple linear regression results showed that pregnant women with electronic screen exposure of more than 5 h per day had higher score of FOC than those with no more than 5 h per day (*β* = 2.02, 95%*CI*: 0.50–3.53, *P* < 0.05). Multiparas had lower scores of FOC than nulliparas (*β*=-1.64, 95%*CI*: -3.27–0.01, *P* < 0.05). Pregnant women without a history of cesarean section had higher score of FOC than pregnant women with a history of cesarean section (*β* = 2.66, 95%*CI*: 0.61–4.71, *P* < 0.05). Pregnant women who experienced likes sour food or hates greasy food had higher score of FOC than those who did not (*β* = 1.75, 95%*CI*: 0.00-3.50, *P* < 0.05). Pregnant women who were not worried about not having a family member with them during delivery had lower score of FOC than those who were worried (*β*=-3.75, 95%*CI*: -5.26–2.25, *P* < 0.001). In addition, pregnant women with good family function (*β*=-0.32, 95%*CI*: -0.64–0.00, *P* < 0.05) and doctor-patient communication (*β*=-0.33, 95%*CI*: -0.64–0.02, *P* < 0.05) had lower score of FOC. However, pregnant women with high anxiety (*β* = 0.50, 95%*CI*: 0.21–0.80, *P* < 0.05) and depression (*β* = 0.30, 95%*CI*: 0.04–0.57, *P* < 0.05) had higher score of FOC. Thus, time of electronic screen exposure per day, parity, history of cesarean section, likes sour food or hates greasy food, worrying about delivery without family members, family function, doctor-patient communication, anxiety and depression can be used to predict FOC in pregnant women in the third trimester (Table 4).

**Table 4:** Results of Multiple linear regression analysis for predicting FOC

Variables	Estimate	95% *CI*		SE	*t*	*P*
		Lower	Upper			
Age (years) (Ref: <35)						
≥ 35	-0.76	-3.12	1.60	1.20	-0.63	0.529
Education (Ref: High school or below)						
College or above	0.47	-1.20	2.15	0.85	0.56	0.579
Self-rated stress (Ref: Big)						
Small	-1.35	-3.31	0.61	1.00	-1.35	0.177
Perceived poor resistance (Ref: Agree)						
Disagree	1.15	-2.42	4.73	1.82	0.63	0.527
Time of electronic screen exposure per day (hours) (Ref: ≤5)						
>5	2.02	0.50	3.53	0.77	2.62	**0.009**
Environmental pollution around the home (Ref: Yes)						
No	-0.70	-2.64	1.23	0.99	-0.71	0.476
Parity (Ref: Nullipara)						
Multipara	-1.64	-3.27	-0.01	0.83	-1.97	**0.049**
History of abortion (Ref: No)						
Yes	-0.19	-1.88	1.51	0.86	-0.22	0.830
History of cesarean section (Ref: Yes)						
No	2.66	0.61	4.71	1.04	2.55	**0.011**
Threatened abortion (Ref: Yes)						
No	-1.51	-3.62	0.59	1.07	-1.42	0.157
Lower abdominal pain (Ref: No)						
Yes	1.79	-0.40	3.97	1.11	1.61	0.108
Vaginal bleeding (Ref: No)						
Yes	0.65	-1.36	2.66	1.02	0.64	0.525
Dizziness and fatigue (Ref: No)						
Yes	1.33	-0.39	3.05	0.88	1.52	0.130
Loss of appetite (Ref: No)						
Yes	0.50	-1.17	2.16	0.85	0.58	0.560
Likes sour food or hates greasy food (Ref: No)						
Yes	1.75	0.00	3.50	0.89	1.97	**0.049**
Worrying about delivery without family members (Ref: Yes)						
No	-3.75	-5.26	-2.25	0.77	-4.90	**< 0.001**
Family function	-0.32	-0.64	0.00	0.16	-1.97	**0.049**
Doctor-patient communication	-0.33	-0.64	-0.02	0.16	-2.10	**0.036**
Social support	0.44	-0.08	0.95	0.26	1.66	0.097
General self-efficacy	0.08	-0.10	0.26	0.09	0.84	0.403
Anxiety	0.50	0.21	0.80	0.15	3.37	**0.001**
Depression	0.30	0.04	0.57	0.14	2.22	**0.027**
Insomnia symptoms	0.08	-0.10	0.26	0.09	0.87	0.387
QOL	-0.23	-0.46	0.01	0.12	-1.87	0.062
Constant	34.52	21.22	47.82	6.77	5.10	< 0.001

**Notes**: QOL: quality of life; Ref: reference; Significant values are in bold

## Discussion

To the best of our knowledge, this is the first cross-sectional study to understand the prevalence of fear of childbirth and its risk factors in a region of China with an intermediate level of economic development in the third trimester of pregnancy. Moreover, in this study, we performed the first analysis of potential risk factors of FOC, including clinical symptoms (e.g., abdominal pain and vaginal bleeding), environmental pollution, and doctor-patient communication. Our study data showed that the overall prevalence of FOC was 56.64%, with 3.93% severe. The prevalence of FOC in this study was slightly lower than the prevalence in those regions from national central cities or economically developed regions of China (Xi’an, Zhengzhou, Chongqing) (range from 67.1 to 70.3%) using the same detection tool (CAQ), but the prevalence of severe FOC was within the range of those regions (range from 2.2–5.5%) [14, 23, 24]. The prevalence of FOC abroad was reported in some previous studies: 4.5% in Belgium, 3.7% in Finland, 24% in Australia, 27% in the United States, and 13.1% in India [13, 44–47]. The prevalence in those countries is significantly lower than our reported, which may be related to the differences of life contexts, ethnic religions, social structures and measurement methods. Therefore, we may conclude that FOC is highly prevalent in China, which requires sufficient attention from academia and medical institutions.

Previous studies found that exposure to electronic screens for more than 5 h per day in pregnant women was a risk factor for depressive symptoms [48]. Moreover, increased exposure to electronic screens was associated with higher depressive symptomatology [49]. Of note, high FOC was associated with using pregnancy-related smartphone applications [24]. Our data showed that electronic screen exposure of more than 5 h per day for pregnant women was a risk factor for FOC. This might be related to pregnant women accessing negative childbirth information on the Internet [50, 51]. Therefore, closer collaboration among media experts, health professionals and policy makers are needed to guide pregnant women to obtain positive childbirth information. We found that high FOC was associated with environmental pollution near the home in the univariate analysis, but further large-sample, multicenter studies are needed to clarify the correlation between environmental pollution and FOC.

In this study, we found that high FOC was associated with nullipara, consistent with several previous studies [9, 14, 52, 53]. It might be due to the lack of experience and information about the childbirth process for nulliparous women [54]. However, some other studies stated that multiparous women experienced FOC with a lower level [13, 55], in which case multiparous women usually experienced traumatic or negative childbirth previously [7]. Notably, in this study, we also found higher FOC scores in pregnant women without history of cesarean section, which is inconsistent with previous findings [13, 56, 57]. This may be related to the fact that the vast majority of young Chinese women who experienced caesarean section previously only consider having a second child if they have a strong desire to have children and are optimistic about childbirth currently. Interestingly, our data showed that pregnant women who like sour food or hate greasy food were more likely to develop FOC. Previous studies showed that pregnant women often experienced altered taste sensations during pregnancy, such as a preference for acidic foods or an aversion to fatty foods [58, 59], which might cause insufficient food diversity. Inadequate food diversity could lead to fetal nutritional abnormalities, and nutritional abnormalities could lead to pregnancy complications and adverse pregnancy outcomes [60, 61]. When faced the upcoming delivery, pregnant women might suffer from FOC due to fear of unhealthiness for themselves and their children. In order to have adequate nutrition for themselves and their fetus, some pregnant women might have to eat foods they didn’t like, adding to the negative emotions that could also be a significant cause of FOC. Therefore, nutrition experts should take measures to ensure a balanced nutritional balance for mother and fetus by reasonably matching foods with full consideration of the pregnant woman’s tastebuds. In addition, our study found that worrying about delivery without family members was a risk factor for FOC. Previous studies showed that the presence and encouragement of family members during childbirth could increase a pregnant woman’s confidence and help her get through the painful process [62, 63]. It is noteworthy that this study took place after the adoption of “Category B” control measures for novel coronavirus infection in China (no more centralized nucleic acid testing, judged close contacts, restrictions on access to public places, centralized isolation, and so on). Pregnant women fear that family members would be infected and need to isolate themselves at home, unable to accompany themselves through the painful process of childbirth or to receive adequate care. Therefore, health care providers need to take into account the concerns of pregnant women and provide timely counseling and quality services.

Our study found that family function was associated with FOC. This finding is similar to the results of several previous studies where inadequate family support was a risk factor for FOC [64, 65]. A possible explanation for this finding might be that family support including information and experiences of childbirth can help pregnant women stay positive about the upcoming childbirth during pregnancy [66]. Previous studies showed that classes for pregnant women taught by midwives, which provide important information on prenatal care and preparation for delivery, can be effective in reducing the prevalence of FOC. However, pregnant women who lack family support may have difficulty accessing such classes, and this may also be an important reason for FOC. Therefore, social workers should communicate with pregnant women’s family members to ensure adequate family support for pregnant women. Our findings suggest that doctor-patient communication was associated with FOC. Previous studies showed that good doctor-patient communication increases patient satisfaction and compliance [67], and that disagreement with the birth plan proposed by the obstetrician was a risk factor for FOC [18]. When a pregnant woman faces a doctor-patient communication dilemma, she may choose not to carry out the doctor’s orders, which may be a potentially important reason for FOC. Therefore, health care facilities need to strengthen training in medical and nursing communication skills to give pregnant women a better health care experience. We also found that anxiety was a risk factor for FOC, which was consistent with previous literature [64, 68]. Since FOC is considered a form of anxiety disorder or a phobic fear [12], it was not surprising that anxiety disorders are associated with higher levels of FOC. In addition, our study also found that depression was a risk factor for FOC, which was consistent with previously literature [13, 23]. Depression, characterized by low mood, lack of pleasure and despair, may lead to negative feelings about childbirth and low self-efficacy, which have been identified as potential causes of fear of childbirth [22, 24, 69]. Therefore, health care providers should focus on counseling for anxious and depressed pregnant women to reduce the prevalence of FOC.

Our study shows that the prevalence of FOC is high and mainly mild. Therefore, effective intervention measures to reduce FOC should be developed. When making the intervention measures, more attention should be paid to the pregnant women who have first birth, no history of caesarean section, and experienced liking sour food or hating greasy food. In addition, reducing electronic screen exposure time, anxiety and depression, and improving family function and doctor-patient communication can also reduce the occurrence of FOC.

There are some limitations of this study that need to be elaborated. First, as a cross-sectional study, causal relationships between variables could not be obtained, and Longitudinal studies are needed in the future. Second, this study was conducted only in a region with a moderate level of economic development in eastern China, which may differ from western, southern, and northern China in terms of life background, ethnic religion, and social structure, and further multicenter studies are needed. Third, FOC, family function, doctor-patient communication, social support, general self-efficacy, anxiety, depression, insomnia symptoms, and quality of life in this study were measured through subjective assessment scales, and lacked objective evaluation indicators, which might cause some bias. Finally, this study only included pregnant women in the third trimester of pregnancy, and pregnant women in the first and second trimester of pregnancy were not included. Further research should be conducted to expand the population in the future.

## Conclusion

In summary, the results revealed that the overall prevalence of FOC was 56.64%, with mild 36.26%, moderate 16.45% and severe 3.93%. It is identified that exposure to electronic screens for more than 5 h per day, Nullipara, History of cesarean section, like sour food or hate greasy food, worrying about delivery without family members, family function, doctor-patient communication, anxiety and depression were significant risk factors in FOC. Considering the high prevalence of FOC, there is an urgent need to develop interventions to reduce the prevalence of FOC in the third trimester of pregnancy, and to pay attention to pregnant women with risk factors for FOC.

## Data Availability

The datasets generated and/or analysed during the current study are not publicly available for ethical reasons but are available from the corresponding author on reasonable request.

## Abbreviations

FOC: fear of childbirth
GDP: gross domestic product
QOL: quality of life
CAQ: Childbirth Attitude Questionnaire
APGAR: Adaptation Partnership Growth and Resolved
CAHPS: Consumer Assessment of Healthcare Providers and Systems
GSES: General Self-Efficacy Scale
GAD-7: Generalized Anxiety Disorder-7
PHQ-9: Patient Health Questionnaire-9
ISI: Insomnia Severity Index
SD: standard deviation
*CI*: confidence interval
SE: standard error
